# The 2023 Turkey Earthquake: Management of 627 Pediatric Musculoskeletal Injuries in the First Month

**DOI:** 10.3390/children10111733

**Published:** 2023-10-26

**Authors:** Mesut Uluöz, Mehmet Yiğit Gökmen

**Affiliations:** Orthopedics and Traumatology Clinic, Adana City Training and Research Hospital, Health Sciences University, Adana 01230, Turkey; mehmetyigit.gokmen@saglik.gov.tr

**Keywords:** earthquake, amputation, disaster medicine, crush injuries, bone fractures

## Abstract

(1) Background: On 6February 2023, two consecutive earthquakes hit Kahramanmaraş and surrounding ten cities, killing over 50,000 people. We aimed to reveal the treatment process of pediatric patients admitted to Adana City Hospital (ACH) in the first month after the earthquake. (2) Methods: Demographic data of the patients, time of presentation to the emergency department, injury locations, treatment procedures, and patient file information were recorded retrospectively and evaluated statistically. (3) Results: There were 1246 patients under the age of 18. A total of 560 patients were hospitalized in the orthopedic clinic; 42% were admitted in the first 24 h and 58% in the first three days. Of these children, 69 (12%) were referred, 52 (10%) were transferred to other departments within the hospital, and 421 (75.2%) were discharged in stable condition. The number of patients with large bone fractures was 77 (34 open fractures). Fasciotomy surgery was performed on 131 patients, 78 of whom had bilateral procedures. Of the 31 patients who underwent amputation, 17 (55%) were performed within the first 24 h and 28 (90%) within the first week. (4) Conclusions: Almost all injured children are admitted to the hospital during the first few days after an earthquake. The management of earthquake injuries in pediatric patients requires specialized care and immediate attention during the treatment process.

## 1. Introduction

The United Nations Office for Disaster Risk Reduction reported that between 2000 and 2019, 552 earthquakes were recorded, which constitutes 3% of the total number of people affected by all disaster types and caused 58% of the deaths [1]. On 6 February 2023, at 04.17 and 13.14, two consecutive earthquakes (Richter 7.8 and 7.6) hit Kahramanmaraş and surrounding ten cities, killing over 50,000 people [2,3]. The early morning timing and the high intensity of the first earthquake, followed by another severe quake in less than nine hours, drastically increased the effects of the disaster. Damaged healthcare facilities in the affected regions have limited the quality and quantity of the service. Most injured survivors had to be transferred to the available centers located in the surrounding cities. Due to damaged roads, transportation via highways was significantly difficult. As a result, air transportation, mainly helicopters, played an important role. As one of the 11 affected cities, Adana was comparatively less affected; 7450 were injured, and 408 lives were lost [4]. All the hospitals in the city were intact, with little to no damage to the staff or equipment. The hospitals began accepting the injured in the city immediately. The Adana City Training and Research Hospital (ACH), with 1575 beds—one of the biggest in terms of bed capacity in Turkey—was equipped with a helipad. The staff was told to suspend all non-urgent elective surgical operations and outpatient clinics. Patients awaiting surgery and those eligible for discharge were released from the hospital. The ACH began receiving earthquake survivors from the severely affected cities several hours after the second quake. Nearly half of the wards were converted into intensive care units, and intensive care capacities were increased. While earthquake victims of all ages were treated, a dedicated block was allocated for pediatric patients, and a joint treatment program was implemented with the pediatric clinic team.

In this study, we aim to demonstrate the orthopedic musculoskeletal injuries resulting from an earthquake and the treatment processes for pediatric earthquake victims, along with the presentation of the difficulties encountered while managing the cases. 

## 2. Materials and Methods

### 2.1. Data Collection 

The study was approved by the Ethical Board of the ACH Hospital on 6 April 2023, with number 2433. The study included a retrospective analysis of the medical records of children who received intervention from the ACH Orthopedics and Traumatology Clinic (OT) between 6 February 2023 and 6 March 2023. The files at the ER with diagnosis codes X39 (exposure to the forces of nature other and undefined) and X34 (earthquake survivor) based on the International Statistical Classification of Diseases and Related Health Problems 10th Revision (ICD-10) were selected. Records of individuals under 18 with one or more orthopedic consultation reports were included in the analysis. Children with internal organ damage and those referred to other centers before orthopedic evaluation were excluded from the analysis. The data included age, gender, time of admission, physical examinations, treatment type, treatment processes, and progress. The surgical approach data included closed reductions, surgical treatment of large bone and/or pelvic fractures, fasciotomies, debridements, closed reduction of large joint dislocations, large bone and joint amputations or disarticulations, small bone and joint amputations, and disarticulation procedures. Debridements performed under anesthesia were classified into three groups based on the number of times they had been performed: once, twice, three times, or more times. 

In addition to the medical treatment data, erythrocyte suspension (ES) and fresh frozen plasma (FFP) usage were recorded. 

### 2.2. Statistical Analysis 

The SPSS (Statistical Package for the Social Sciences) 26.0 package program was used for statistical analysis of the data. Categorical measurements were summarized as numbers and percentages. 

### 2.3. Ethics 

The study adhered to the Declaration of Helsinki guidelines and was approved by the Adana City Training and Research Hospital Clinical Research Ethics Committee (Meeting number: 123, Decision number: 2433, 6 April 2023). 

## 3. Results 

**Figure 1:**
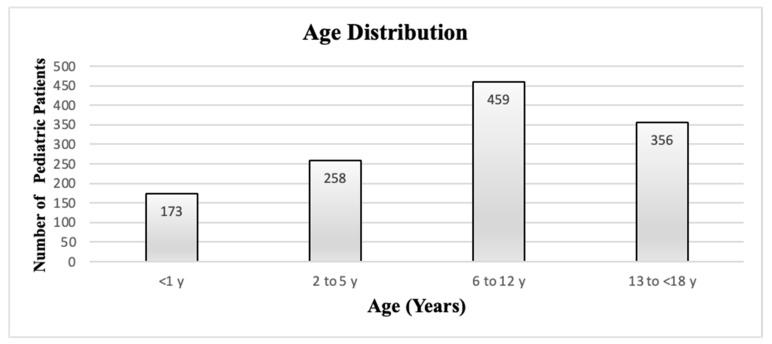
Age distribution of pediatric patients admitted.

A total of 4586 patients were admitted to our emergency department with the diagnoses of ICD-10 X34 (earthquake victim/earthquake survivor) and ICD-10 X39 (exposure to the forces of nature, other and unspecified) in one month from the day of the two major earthquakes. There were 1246 (635 males and 611 females) patients under 18. The age distribution of pediatric patients admitted to the emergency department in one month is presented in Figure 1. 

**Figure 2:**
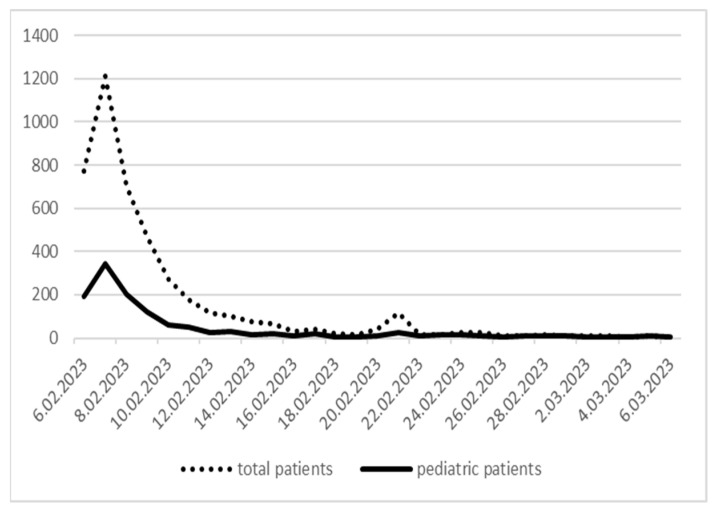
The daily distribution of the number of earthquake victims who came to the ER in one month.

In total, 42% were admitted in the first 24 h, 58% in the first three days, and 80% in the first week. The daily distribution of the number of earthquake victims who came to the emergency department in the first month is presented in Figure 2. 

Orthopedic consultation was requested for 627 (50.32%) underage earthquake victims. 

The initial orthopedic approach was conducted by an orthopedic team consisting of a lecturer, an orthopedist, and two residents of the orthopedic clinic. Following the physical examinations, 560 (n = 298 male, n = 262 female, and mean age = 9.94 ± 5.27) of the 627 were hospitalized and treated by the orthopedic clinic.

Surgical treatment was applied to 344 patients (61.43%) with musculoskeletal injuries: extremity crush injuries with acute compartment syndrome, large bone and/or pelvic fractures, large bone amputations, and debridements for wounds, lacerations, and foreign body penetrations.

Patients with symptoms of paralysis, paresthesia, and pulselessness, which indicated acute compartment syndrome (n = 131, 62.68%), underwent emergency fasciotomy surgery. The distribution of the fasciotomy localizations for upper extremity, lower extremity, and both was 41, 90, and 8, respectively. Among the fasciotomy cases, hospitalization for observation was the preferred approach for individuals who did not have a crush injury but still showed symptoms of compartment syndrome. A total of 48 patients who underwent fasciotomy surgery were also operated on for extremity fractures. 

The initial interventions, including closed reduction, splinting, and Velpeau bandaging, for all fracture cases (n = 238) were conducted in the trauma rooms of the emergency clinic. In 161 cases (67.65%), conservative fracture treatment was performed. The majority (n = 102, 42.86%) had upper extremity fractures. 

The number of patients with large bone and/or pelvic fractures who underwent surgery included 46 males and 31 females, with a mean age of 10.31 ± 4.95 years. The majority of open fracture cases, which amounted to 34, were located at the lower extremities (n = 26). In the treatment of open fractures, external fixators and K-wires were used. 

In six cases, multiple extremity fractures were observed, including three cases with pelvis and femur fractures, two with tibia and femur fractures, and one case with humerus and femur fractures. 

Pelvic ring fractures were present in seven children, including three iliac crest fractures, two open book fractures, and one vertical shear injury. 

The distribution of fractures in surgically treated patients, according to anatomical site, is given in Table 1. 

During the management of the injured children admitted to the orthopedics ward, 69 (12%) were referred, 52 (10%) were transferred to other departments within the hospital, 421 (75.2%) were discharged in stable conditions, and 18 (3%) were lost.

A total of 284 pediatric patients received blood products (915 units of erythrocyte suspension and 318 units of fresh frozen plasma). 

Amputation was performed on 31 patients, two of whom underwent minor amputations. More than half of the major amputations were performed (n = 17, 55%) within the first 24 h of admission, and nearly all (n = 28, 90%) were completed within the first week. 

The number and distribution of orthopedic procedures, fasciotomies, and amputations performed are given in Table 2.

## 4. Discussion 

In the first few days, the main challenges were the increasing number of injured people in the ER and the lack of a quick response. Previous studies suggest that ER patient density may increase to 66% in the first 24 h after an earthquake [5,6]. The first few hours were especially misleading in predicting the number of injured people since the number of admissions quickly rose after the first day. The lower-than-expected number of patients on the first day might be attributed to the slow start of debris removal. There were further delays due to damaged roads and a lack of transportation options. Patients arrived throughout the first week after the earthquake. 

The current study was carried out to document the total impact of the earthquake on children. To do this, we suggest evaluating patient flow and problems at each stage. 

The first stage lasted for three days after the earthquake. The main issue was the accumulation of patients in the emergency and operating rooms. There was no shortage of consumables at this stage. Hospital staff was shifted to patient admission, triage, and ER, and wards were converted into intensive care units (ICU), which caused shortages in other areas and adversely affected overall care. All operating room staff worked 12-h shifts coordinated by orthopedists. Many hospital staff did not leave the hospital in the first week, and their families joined them there since their houses were damaged and they feared aftershocks. They spent their resting time in their cars and tents. Although there are existing guidelines for all kinds of disasters, the occurrence of two consecutive massive earthquakes within approximately nine hours dramatically increased the damaging effect on both people and healthcare structures, resulting in an unprecedented crisis.

At the end of the third day, burnout started among emergency and operating room personnel. If there had been places for personnel to rest, they would have had better physical and mental endurance. Equipment and consumables were readily available, but there was a shortage of surgeons and operating room nurses. On the third day, volunteer orthopedists from other cities helped relieve the orthopedic team. It took a week for volunteer nurses and other health personnel to arrive. At the end of the first week, the team was large enough for full-time work, but the density of patients in the operating room had already decreased. Since nearly all ER admissions occurred within the first week, we believe that this type of personnel and medical device support strategy should be planned in earthquake contingency plans [7,8]. 

Since the earthquake occurred during the winter, there was a threat of hypothermia, a significant issue in natural disasters, as emphasized by Oshiro et al. [9]. Patients were brought to the ER with heat-insulating blankets. However, because they had been exposed to cold for a long time, especially the lower extremities, they were still cold, and there were neurovascular problems, even in patients with no signs of trauma. These problems gradually improved in most patients as they were warmed under appropriate and controlled conditions. 

Various injuries can occur in earthquakes, with musculoskeletal injuries being the most prevalent [7,10]. Due to the fatal impact of the injuries to the head, abdomen, and chest, most die before they can be reached [11]. In our study, approximately 50% of the patients had musculoskeletal injuries. MacKenzie et al.’s review of earthquake injuries reports that fractures are most common in the tibia; however, in our study, it was the femur [12]. This review indicates that two-thirds of orthopedic injuries were fractures; in our study, it was nearly half. We attribute the low fracture rate to the ability of children to fit more easily in small spaces under debris. 

The hospital where our study was conducted is not a field hospital. It is the largest third-level hospital in the region. Among the hospitals in the earthquake area, those in serious condition were referred to our hospital, which explains the high rate of open fractures and large bone fracture surgeries.

Open fractures occur when bone fragments break the skin from the inside out. In earthquake injuries, debris hits the body and breaks bones by cutting through the skin from the outside. This type of open fracture is often contaminated and can worsen if patients spend time trapped or are slow to arrive at the hospital [13]. A few patients’ anamneses include fractures that closed after the earthquake and were re-opened by rescuers who pulled them out from under the debris. This underscores the importance of careful rescue work. 

Due to the high rate of open fracture and compartment syndrome in the surgical treatment of large bone fractures, we mainly used external fixation. This is a routine practice in earthquake injuries and requires the use of C-arm fluoroscopy. Some authors report difficulties in surgery due to the inaccessibility of fluoroscopy [11]. The three fluoroscopy devices in our operating room were insufficient for the number of patients in the first few days, so patients were queued based on urgency. In the meantime, injuries were splinted, and treatment was started in the ward. When three more fluoroscopy devices arrived from other cities, this problem disappeared. With more devices, we were able to apply a damage control orthopedics strategy similar to that used for multiple-trauma patients. Morelli et al. emphasized that this strategy is effective in reducing mortality and complications [14]. Compartment syndrome is frequently associated with crushing injuries [15]. In the 2010 Haiti earthquake, fasciotomies were not performed because compartment cases were chronic [11]. Some authors suggest that fasciotomies performed in chronic cases increase infection and amputation rates [16]. In our study, most patients who underwent fasciotomies had acute compartment syndrome, which required emergency fasciotomies. 

Extremity salvage was applied, and fasciotomies were performed when it was unclear if a fasciotomy or amputation was most appropriate. Unfortunately, this resulted in amputation for most patients, which increased the workload due to the need for debridement. We believe this happened because many children’s families were absent and it was difficult to find informed consent. 

Fasciotomy is an extremity salvage procedure for acute compartment syndrome. Without meticulous wound care, serious infections can develop after compromising skin integrity [16,17,18]. Patients with wounds showing superficial necrosis who did not undergo fasciotomy were also subjected to debridement. However, in our study, the majority of debridement procedures were performed in fasciotomy areas.

Patients with fasciotomies received daily visits and dressings. Especially in young children, bedside debridement, dressings, and local anesthesia were not possible. Instead, this was carried out under operating room conditions. In some cases, bedside debridement was performed after an anesthesiologist sedated the patient. Hence, there was a need for anesthesiologists and anesthesia technicians, partially met by volunteer anesthesia teams from other cities. Wound debridement was the most frequently performed orthopedic procedure, a trend observed in many similar studies, including Morelli et al.’s review of pediatric earthquake patients between 1999 and 2014 [14]. 

After ten days, the second phase started, and service intensity increased. At this stage, signs of infection began to appear in patients who underwent fasciotomies. Daily dressings were made with paraffin-antiseptic tulle gauze. There were shortages of people on dressing teams and in consumables, especially saline and antibiotics. This was anticipated, and consumables were brought from other regions. 

Wound cultures were obtained in 77 pediatric patients who underwent orthopedic surgical treatment due to surgical site infection. Microbial growth was detected in 44 (57%) patients. Surgical site infection and growth in cultures were seen in 36 patients who underwent fasciotomy surgery. The wound samples were isolated from non-fermenting Gram-negative bacilli, Gram-positive cocci, Enterobacteriaceae, and yeast-like fungi. *Acinetobacter* spp., *Pseudomonas aeruginosa*, *methicillin-resistant Staphylococcus aureus, Serratia marcescens, Klebsiella pneumoniae, Citrobacter koseri*, and *Candida albicans* are the major bacterial isolates of wound infections. Pediatricians arranged appropriate antibiotics. When the pathogens grown in cultures are evaluated, it is seen that they are compatible with the pathogens in similar studies in the literature [19]. 

Although we paid great attention to sterility in fasciotomy sites, appropriate antibiotic therapy, and conducting frequent debridement and wound care, we could not prevent infection or muscle necrosis in some patients. For these cases, we applied VAC (Vacuum-Assisted Closure) and/or hyperbaric oxygen therapy. Due to the high number of patients, the VAC device could be used by about half of the patients. After the wound was covered with a VAC drape, the remaining patients were connected to bedside aspirator systems until the VAC device arrived. In total, 83 children received VAC therapy. Wound dressings were changed every three days, and an average of five sessions were applied.

Hyperbaric oxygen therapy was used both to fight infections and to treat extremities with questionable circulation. Hyperbaric oxygen therapy was administered to 67 patients in one-hour daily sessions. Successful hyperbaric oxygen therapy has been reported for soft tissue injuries, open fractures, and crush injuries [20,21]. The availability of a hyperbaric oxygen center in our hospital was a marked advantage for inpatient treatment. 

Some studies report that hyperbaric oxygen therapy has a neuroprotective effect in the treatment of neurological injuries [22]. Therefore, we also used hyperbaric oxygen therapy for nerve injuries. Although there is no clear information about its efficacy, we mobilized all our resources. In our study, radial and peroneal nerve injuries were prominent. Similar to Dai et al.’s study, all radial nerve injuries were associated with humeral fractures [23]. Nearly half of patients with peroneal nerve injuries had no sign of trauma in their lower extremities. These patients could do plantar flexion but not dorsiflexion. All patients recovered within ten days with vitamin B12 and hyperbaric oxygen therapy. We think that this nontraumatic peroneal nerve injury is caused by the temporary deterioration of nutrition in the anterior compartment of the leg when exposed to cold for a long time. However, we could not find any information in the literature to support this idea, so this is only our supposition. Since this patient group was discharged quickly, no study, such as EMG checks after three weeks or imaging of the anterior leg compartment, could be conducted. The third phase started at the beginning of the third week, when we began closing fasciotomies. This process was carried out with the plastic surgery team. The anticipated shortage of plastic surgeons was addressed with volunteers. There were no problems at this stage. If extremity fasciotomies had softened enough to approach the edges, they were closed either gradually or in a single session. Skin grafts were used when necessary. 

In our study, extremity injuries such as large bone fractures and major amputations led to high mortality and morbidity. The most common cause of amputations was crushing injuries. Of the 31 patients who underwent amputation, 17 (55%) were performed within the first 24 h and 28 (90%) within the first week. This reflects the seriousness of the injuries. In addition, seven patients lost two limbs at the same time, and six of them were treated within 24 h. This rate of amputation is higher than those in the literature since severe patients were referred to our hospital, including those requiring large bone fracture surgery. The majority of amputations performed in the first week after the earthquake were total or subtotal traumatic amputations. A small number were due to infection and subsequent necrosis, as shown in the study by Bar-On et al. [11]. 

When children are referred, it is important to obtain a patient contact record since it simplifies follow-up after discharge. Otherwise, rehabilitation and preparation of prosthetics are delayed. Pediatric patients need an absolute companion, especially when dealing with amputation, bone fracture surgery, and immobilization due to compartment syndrome. Nearly half of the patients did not have family presence. They were accompanied by female volunteers, mostly teachers. This was important for the care and safety of the children. DNA samples were taken in an attempt to reach their families. 

The psychological impairment added to the physical injuries of child earthquake victims affected compliance due to poor communication. The pediatric psychiatrists were consulted, and the majority of the children received psychosocial rehabilitation. Efendi et al. reported that acute stress disorder was more prevalent among earthquake survivors, particularly in children with no history of psychological trauma. The severity of the trauma was higher in young people who had lost parents or close relatives [24]. It is important to remember that children's emotional and social needs may be neglected during the chaotic aftermath of an earthquake. Based on early research, it is recommended that steps be taken to integrate psychosocial support into the treatment protocol beforehand. 

One of the most important problems we encountered was malnutrition, as in similar situations where children lacked protein, energy, iron, and trace elements [25]. Post-traumatic stress disorder (PTSD) may have been caused by poor nutrition. We tried to feed children a protein-rich diet with the help of the dietitian team in our hospital. We would have been more successful if there had been a team to help prevent PTSD, as stated in Kikuchi et al.’s study [26]. 

Both malnutrition and open wounds cause anemia and hypoalbuminemia [27]. The need for intensive care increased the need for blood products. On average, three erythrocyte suspensions (ES) and one fresh frozen plasma (FFP) were used per pediatric patient. This ratio can improve contingency plans for earthquakes. Early rehabilitation was started by the physical therapy clinic. 

The literature suggests that rehabilitation should be started immediately after the acute stage of trauma for the continuation of functionality [28]. 

The final stage was the discharge of patients who had recovered. By this time, most patients no longer had a home or family, which made discharge difficult. We suggest that contingency plans should designate youth treatment centers or residences to receive discharged patients. This would help free up space for arriving patients. 

This study has major limitations. First, this was a retrospective study based on medical records. The accuracy of patient digital files may have been compromised under crisis conditions, although many of them were double-checked. In addition, this study focused on musculoskeletal injuries in earthquake survivors admitted to ACH. This study did not report details of major organ injuries or additional conditions. 

All hospital staff made devoted efforts to help earthquake victims. We think it would be helpful for non-orthopedic workers to know the basic orthopedic approach for such situations. It would also be helpful if patients’ families took a more active role in follow-ups. 

## 5. Conclusions 

Severe earthquakes create chaotic conditions in the delivery of care. Particularly, hospital staff in neighboring provinces that were relatively less affected by the quake should promptly get to an alert level, and emergency clinic and operating room preparations should be completed as soon as possible. Hospital bed capacity should be increased, and patients capable of home treatment should be discharged immediately to free up beds. The shortages of consumables (antibiotics, saline, dressing materials, orthopedic implants, etc.), fluoroscopy devices, and additional personnel should be addressed right after the earthquake, preferably by the end of the first day. 

The treatment of earthquake-affected children requires a multidisciplinary approach and careful planning. Surgeons, anesthesiologists, pediatricians, physiotherapists, child psychiatrists, and dietitians should work in an organized manner and coordinate with local authorities and security forces. The management of earthquake injuries in pediatric patients requires special attention during the treatment process. In particular, nutrition, safety, and psychosocial support are more important in the recovery process than managing adult patients. 

## Figures and Tables

**Table 1:** The distribution of fractures in surgically treated patients according to anatomical site.

Localization	*n*	%
Upper-limb fractures	18	23.4%
Humerus	14	18.2%
Diaphysis	9	11.7%
Epiphysis	4	5.2%
Neck	1	1.3%
Radius/Ulna	4	5.2%
Lower-limb fractures	52	67.6%
Femur	31	40.3%
Diaphysis	25	32.5%
Epiphysis	2	2.6%
Neck	4	5.2%
Tibia/Fibula	21	27.3%
Pelvis	7	9%

**Table 2:** The number and distribution of orthopedic procedures.

Orthopedic Procedures	*n*	%
Fasciotomies	131 (78 bilateral)	38.1%
Debridements	91	26.5%
once	18	
twice	41	
three times and more	32	
Closed reduction of large joint dislocations	14	4%
Shoulder	9	
Elbow	3	
Hip	2	
Major amputations or disarticulations	29	8.4%
Minor amputatitons or disarticulations	2	0.6%
Surgical treatment of large-bone fractures	70	20.4%
Surgical treatment of pelvic fractures	7	2%

## Data Availability

The data used in this study comprise the de-identified patient records saved in the ACH medical records archive, to which regulations block open access. Permission for this research is limited to the researchers exclusively. Nevertheless, availability to third parties might be upon plausible request provided to the ACH Ethical Committee.
